# A biphasic nanohydroxyapatite/calcium sulphate carrier containing Rifampicin and Isoniazid for local delivery gives sustained and effective antibiotic release and prevents biofilm formation

**DOI:** 10.1038/s41598-020-70726-3

**Published:** 2020-08-24

**Authors:** Irfan Qayoom, Rahul Verma, Prem Anand Murugan, Deepak Bushan Raina, Arun Kumar Teotia, Saravanan Matheshwaran, Nisanth N. Nair, Magnus Tägil, Lars Lidgren, Ashok Kumar

**Affiliations:** 1 Department of Biological Science and Bioengineering, Indian Institute of Technology Kanpur, Kanpur, UP 208016 India; 2 Centre for Environmental Sciences and Engineering, Indian Institute of Technology Kanpur, Kanpur, UP 208016 India; 3 Centre for Nanosciences, Indian Institute of Technology Kanpur, Kanpur, UP 208016 India; 4 Department of Chemistry, Indian Institute of Technology Kanpur, Kanpur, UP 208016 India; 5 Department of Orthopaedics, The Medical Faculty, Clinical Sciences Lund, Lund University, Lund, Sweden

**Keywords:** Drug delivery, Chemical biology, Microbiology

## Abstract

Long term multiple systemic antibiotics form the cornerstone in the treatment of bone and joint tuberculosis, often combined with local surgical eradication. Implanted carriers for local drug delivery have recently been introduced to overcome some of the limitations associated with conventional treatment strategies. In this study, we used a calcium sulphate hemihydrate (CSH)/nanohydroxyapatite (nHAP) based nanocement (NC) biomaterial as a void filler as well as a local delivery carrier of two standard of care tuberculosis drugs, Rifampicin (RFP) and Isoniazid (INH). We observed that the antibiotics showed different release patterns where INH showed a burst release of 67% and 100% release alone and in combination within one week, respectively whereas RFP showed sustained release of 42% and 49% release alone and in combination over a period of 12 weeks, respectively indicating different possible interactions of antibiotics with nHAP. The interactions were studied using computational methodology, which showed that the binding energy of nHAP with RFP was 148 kcal/mol and INH was 11 kcal/mol, thus varying substantially resulting in RFP being retained in the nHAP matrix. Our findings suggest that a biphasic ceramic based drug delivery system could be a promising treatment alternative to bone and joint TB.

## Introduction

Tuberculosis (TB) is a global disease that caused an estimated 1.2 million deaths in 2018 and around 10 million new TB cases were reported globally in the same year, with the incidence being stable in the recent years as per World Health Organization (WHO) report^1^ Osteoarticular tuberculosis is a common manifestation accounting for 10–15% of all extrapulmonary TB (EPTB)^2,3^ cases and 1–4% of all TB cases^4–6^. The bone and joint TB is most often found in spine and weight bearing extremities resulting in neurological complications and joint destruction^7^. Treatment modalities include surgical debridement of bone lesions, instrumental stabilisation and filling of dead space with allografts or autograft as well as systemic administration of anti-tuberculosis drugs (ATDs) like Isoniazid (INH), Rifampicin (RFP), Pyrazinamide (PZA), Ethambutol (EMB) and Streptomycin (SM). However, multiple drug resistance and TB recurrence has made it necessary to adhere to long-term, systemic oral use of multiple anti-tuberculosis drugs^8,9^. Long-term systemic administration of first line drugs such as RFP and INH is still limited by low bioavailability at the bone nidus due to poor tissue penetration^10,11^. Side effects of systemic use include nausea, vomiting, hepatotoxicity, deafness or vestibular dysfunction^12^ makes clinicians prone to resort to second line drugs, less toxic but also less clinically efficient. To circumvent the draw backs, there has been a paradigm shift towards development of biomaterials that will act as carriers of drugs. Local drug delivery in a bone cavity has dual purposes, one to deliver the drugs in a sustained and controlled manner to prevent the chances of reinfection, and secondly to enhance bone healing as a bone filler substitute.

To achieve the goal of drug delivery, an array of different biomaterial based drug delivery systems (DDS) have been designed^9,13–15^. However, the incessant local delivery of single ATD could increase the propensity of drug resistant strains that might enhance the chances of relapse of infection^15^. Moreover, biofilm formation on the hydrated matrix secreted by the pathogen on the inert surface of implants is the primary cause of implant rejection and revision surgery. Pathogens embedded in biofilms have been shown to act as a barrier to the penetration of antibiotics thus reducing the antibiotic susceptibility compared to their planktonic counterparts^16^. This leads to persistent or recurrent infections and thus forcing the clinicians to resort to revision arthroplasty^17,18^.

It has been already established that anti-catabolic drugs, bisphosphonates, show potent inhibition of bone resorption activity by binding strongly to the hydroxyapatite crystals^19^. Moreover, intensive efforts have been put to understand the binding mechanisms of different drugs and proteins occurring at the surface of hydroxyapatite^20^. Computational methods using modelling and simulations are powerful tools to study the structural characteristics of drugs/proteins and bone mineral hydroxyapatite and interactions between them^21^.

The aim of this study was to use a biphasic CSH/nHAP based nanocement (NC) carrier for local delivery of two first line ATDs (RFP and INH) to achieve the long-term sustained and controlled release. Similar carriers have been previously used as a carrier for bone morphogenetic proteins (BMP) and zoledronic acid (ZA) to promote either the fracture healing^22–24^ or enhance the bone mineralization in osteoporotic rats^25,26^. To our knowledge, this study is the first to describe the release profile of tuberculosis drugs in a ceramic carrier. Furthermore, we aimed to evaluate the interaction between the two ATDs with nHAP. Finally, as a proof of concept, we used an in-vitro biofilm model and evaluated the antibacterial and antibiofilm effect of the drugs released from the carrier. We also created an in-vitro infection model to study the elimination of intracellular pathogen in infected macrophages. It was hypothesized that the resorbable calcium sulphate phase will provide an initial burst release of both the drugs while RFP or INH could also chemically interact with nHAP to provide a sustained release of the drugs over a longer period of time. By achieving this differential release pattern of the added drugs, it might be possible to achieve drug concentrations several times higher than the minimum inhibitory concentration (MIC) or minimum biofilm eradication concentration (MBEC) to initially inhibit the biofilm quorum while the second phase of drug release could potentially eradicate any remaining bacteria in the vicinity.

## Materials and methods

### Materials

Calcium nitrate and diammonium hydrogen phosphate were purchased from SD Fine-Chem, India. Minimal essential media-alpha (α-MEM), 3-(4,5-dimethylthiazol-2-yl)-2,5-diphenyltetrazolium bromide (MTT) and trypsin–EDTA were all purchased from Sigma Chemical Co. (St. Louis, MO, USA). Fetal bovine serum (FBS) and phenol red free α-MEM were purchased from Gibco (Waltham, MA, USA). Antibiotic cocktail (penicillin and streptomycin) was purchased from Hi-Media, India. Rifampicin and Isoniazid were purchased from Merck (Darmstad, Germany). All other chemicals used were of analytical grade.

### Preparation and characterisation of nanocement (NC) and drug impregnated NC (drug@NC)

To prepare nanocement matrix, first nanohydroxyapatite (nHAP) and calcium sulphate hemihydrate (CSH) were synthesized as described previously^22^. NC matrix was generated by mixing nHAP and CSH in a ratio of 40:60 to which 600 μL/g of liquid was added and the slurry was allowed to set for 15 min. To incorporate the RFP and INH in the nanocement, a gradient dose of RFP (1%, 3.3%, 5% & 10%) and INH (1%, 1.65%, 5% & 10%) were mixed with NC powder prior to the addition of solvent (600 μL/g). The different dosages (3.3% RFP & 1.65% INH) were used for evaluating the release kinetics and cell material interactions based on clinical dosages of RFP (600 mg/kg) and INH (300 mg/kg) and considering commercial bone filler CERAMENT (18 g/unit). The subsequent solid discs were further characterised by scanning electron microscopy (SEM) (ZEISS, Germany) to evaluate any gross changes in the microstructure of nanocement upon addition of drugs. Further, the drug@NC (both RFP and INH) were evaluated for changes in the setting time (n = 3 per sample) using Vicat needle test apparatus (AIMIL LTD., India) and mechanical properties (compressive strength) (n = 5 per sample) using universal testing machine (INSTRON) at a constant load and displacement of 100 kN and 0.6 mm/min, respectively.

### In-vitro drug release profile from drug@NC

To evaluate the release profile of INH and RFP from the NC matrix, different ratios of CSH:nHAP: **Nanocement1** (**NC1)-80:20; Nanocement2 (NC2) – 60:40 & Nanocement (NC3) – 40:60** were used to prepare the solid nanocement discs to which 1.65% (w/w) INH and 3.3% (w/w) RFP alone or in combination were mixed prior to the addition of solvent. The cylindrical discs of size 10 mm height × 5 mm diameter were immersed in 1 mL phosphate buffered saline (PBS) and incubated at 37 °C. At 0.25, 1, 3, 7, 14, 21, 28, 42, 56, 70 & 84 days, the whole PBS was removed for measuring the absorbance and fresh 1 mL PBS was added instantly. The drug concentration, as percentage cumulative release, was calculated from the absorbance measured for RFP and INH at 474 nm and 262 nm, respectively using spectrophotometer.

### Nanohydroxyapatite—Drug Interactions

To evaluate the interaction of nHAP with RFP and INH, we performed fourier transform infrared spectroscopy (IR) and computational studies.

### Fourier-transform infrared spectroscopy (FTIR)

The nHAP powder was mixed with RFP and INH in a ratio of 1:1 to which 600 µL/g water was added followed by drying in hot air oven to determine the FTIR spectra (Spectrum Two, PerkinElmer) within the range of 4000 cm^−1^–400 cm^−1^.

### Computational studies

In order to understand the adsorption of INH and RFP on nHAP, we carried out computational studies and obtained adsorption structures and adsorption energies. We constructed a model for the (010) termination of nHAP which corresponds to one of the most exposed surfaces of nHAP under physiological conditions^27^. In particular, we chose the hydroxylated surface model of (010) nHAP at pH≈7 based on the report by *Lin and Heinz*^28^. An orthorhombic supercell was constructed based on the experimental cell parameters a = 9.3 Å, b = 9.3 Å, c = 6.9 Å, α = β = 90°, γ = 120°^29^. The size of the supercell is 27.50 × 37.66 × 50.25 Å^3^, and contains 320 Ca, 256 P, 1088 O, and 104 H atoms. A vacuum space of 20 Å was created above the surface along the Z-direction. Structure optimization of the whole system was then carried out (with fixed cell parameters) using the Material Studio program^30^ package employing the COMPASS force field^31^.

The RFP and INH structures were initially optimized in vacuum. The optimized drug molecule (RFP/INH) was then adsorbed on the optimized nHAP (010) hydroxylated surface in two independent calculations. The COMPASS force field^31^ was used to describe the intramolecular and intermolecular interactions of the drug molecule and nHAP. Canonical ensemble molecular dynamics (MD) simulations at 300 K were carried out using the Forcite module of Material Studio by relaxing only the drug molecules. We employed the Nosé thermostat in these simulations^32^.

Different initial orientations of the drug molecules were adsorbed on the nHAP surface and MD simulations were performed for each case for 50 ps with a time step of 1 fs. A total of 100 initial low-energy configurations were chosen from these trajectories to carry out structure optimizations. During these optimizations, the adsorbate and the whole nHAP atoms were relaxed. Out of these 100 structures, 13 (10) low energy ones lying within a binding energy window of 1 kcal/mol in the case of RFP or INH were further analysed.

The binding energy ΔE was computed as,

$$\mathrm{\Delta E}=(\mathrm{EHAP}+\mathrm{ ED}) -\mathrm{ Ecomplex}$$

where Ecomplex is the total energy of nHAP-RFP or nHAP-INH systems, EHAP and ED are the total energies of nHAP surface and RFP/INH molecules, respectively.

We have also computed the IR spectra of the bare surface, adsorbates in gas-phase, and adsorbed states. They were computed by taking the Fourier transform of the dipole autocorrelation calculated along the NVT ensemble trajectories of the energetically most stable conformations. For RFP/INH, nHAP surface, and nHAP-RFP/INH systems, we have taken 20 ps, 500 ps, and 500 ps long trajectories, respectively, for this analysis.

### Kirby-Bauer (KB) disk diffusion assay

This assay was performed to evaluate the antimycobacterial activity of released pristine RFP and INH from nanocement by measuring the zone of inhibition (in mm) around the NC and drug@NC pellets (gradient concentrations described above). Briefly, the *Mycobacterium smegmatis* was grown till mid-log phase in Middlebrook broth supplemented with 10% oleic acid, albumin, dextrose, catalase (OADC) and 0.25% Tween 20 was added to prevent agglomeration of the bacilli. The *Mycobacterium smegmatis* (100 µl culture) was consequently spread on the surface of 7H12 Middlebrook agar plates supplemented with OADC. This was followed by placing of NC pellets and drug@NC pellets (1 cm diameter × 1 mm height) of gradient concentration on the surface of agar plates in triplicates. The plates were allowed to grow by incubating at 37 °C for 72 h after which the zone of inhibition was calculated by measuring the diameter of the clear zone using a digital Vernier calliper.

### Liquid inoculation assay

The *Mycobacterium smegmatis* was first allowed to grow till mid-log phase in 7H12 Middlebrook broth supplemented with growth supplement 10% OADC and 0.25% tween 20 was added to hamper the agglomeration of the bacteria. The cylindrical NC and drug@NC discs of size 10 mm height × 6 mm diameter were prepared and soaked in the corresponding test tubes wherein 10 mL of broth was added and inoculated with 100 µl culture of *Mycobacterium smegmatis*. After 72 h, the grown cultures were used to determine the colony forming units (CFUs) by serially diluting in broth and then plating them on agar plates.

### Resazurin assay

Resazurin assay was used to assess the antimycobacterial activity of released pristine RFP and INH from NC. The NC and drug@NC beds were prepared in 96-well plate using gradient concentration of each drug alone and in combination (as described above). The wells were inoculated with *Mycobacterium smegmatis* in 7H12 Middlebrook broth supplemented with 10% OADC and 0.25% Tween 20 and the plates were incubated for 72 h at 37 °C. Resazurin at final concentration of 0.0015% was added into each well and incubated for 24 h at 37 °C in fresh 96-well plate. After incubation, the florescent intensities were measured at an excitation wavelength of 540 nm and emission wavelength of 590 nm using Thermo Scientific VARIOSKAN FLASH MULTIMODE READER. The percentage reduction of resazurin was calculated using the below formula:

$$\%Reduction=\frac{FlTest}{FlCtrl} \times 100$$

where Fl_Test_ = Florescent intensity of the test, Fl_Ctrl_ = Florescent intensity of broth control.

### Antibiofilm assay

The NC and drug@NC pellets were evaluated for inhibiting the biofilm formation by three reference bacteria, *Mycobacterium smegmatis, Staphlococcus aureus* and *Bacillus subtilis* in their respective media with several components as an inducer of biofilm formation. Briefly, the NC and drug@NC (3.3% RFP & 1.65% INH alone and in combination) pellets of size 6 mm diameter × 2 mm height were placed in 24 well plates. The wells were inoculated with 1% of mid log phase of bacteria in broth culture. For biofilm formation, *S. aureus* and *B. subtilis* were incubated at 37 °C for 3 days in tryptic soy broth (TSB) while as for *M. smegmatis,* M63 media supplemented with 0.5% casamino acids, 1 mM magnesium sulphate and 0.7 mM calcium chloride was used. The cultures were incubated at 37 °C for 7 days without any disturbance in static condition. After biofilm formation, the wells were observed visually for the formation of biofilm and the pellets were taken out carefully, washed with PBS gently and vacuum dried for electron microscopy (SEM) (ZEISS, Germany) while the plates were stored for crystal violet assay. Furthermore, the *M. smegmatis* was transformed with GFP expressing PTiGC vector and allowed to form biofilm on the surface of NC and drug@NC. The inhibition of biofilm by the drug impregnated NC was determined by Confocal Laser Scanning Microscopy (CLSM) (Leica) producing 3D images of biofilm as well.

### Cell material interactions

The biocompatibility and cytocompatibility of NC and drug@NC (3.3% RFP & 1.65% INH alone or in combination) was assessed by culturing murine pre-osteoblast MC3T3E1 cells on the surface of pellets (1 × 10^5^ cells/pellet). The cell viability was evaluated by MTT assay as described elsewhere^24^. Cell adhesion, cell appendages and cell spreading was determined by SEM (ZEISS, Germany) and Confocal Laser Scanning Microscopy (CLSM) (LEICA). For CLSM, the cultured cells were fixed with 4% paraformaldehyde (PFA) and permeabilized with 1% TritonX-100 followed by staining with blue 4′,6-diamidino-2-phenylindole (DAPI) for nuclei.

### In-vitro Mycobacterium smegmatis infection

To investigate the intracellular elimination and killing of pathogen, RAW264.7 murine macrophages were grown on NC and drug@NC pellet beds fabricated in 24 well plates using DMEM supplemented with 10% FBS and 1% antibiotic cocktail. After 24 h, the seeded cells were infected with *M. smegmatis* (mid-log phase) at a multiplicity of infection (MOI) of 1:10 for 4 h using DMEM supplemented with 10% FBS devoid of antibiotics. After 4 h, the infected cells were incubated for 1 h in 1 × PBS containing 1% penicillin–streptomycin antibiotic cocktail to kill all remaining extracellular pathogens. The cells were grown for 24 h in DMEM containing 10% FBS and 1% antibiotic cocktail and then harvested and lysed in 0.1% TritonX-100 for 30 min. The cell lysate was plated on 7H12 Middlebrook agar plates after serial dilution to count the CFU. For microscopic visualization in CLSM, the *M. smegmatis* was transformed with PTiGC vector that has a constitutive green florescence protein (GFP) expression. The RAW 264.7 cells seeded on NC and drug@NC were then infected with transformed *M. smegmatis* after 24 h. Post-infection the cells were fixed in 4% PFA for 30 min and permeabilized with 1% TritonX-100 followed by visualization in CLSM. To determine the successful infection of macrophages, RAW264.7 cells were also seeded on plasma cleaned circular coverslips and infected with transformed *M. smegmatis* followed by staining with red florescent phalloidin-tetramethylrhodamine B isothiocyanate (TRITC-phalloidin) for cytoskeleton and blue 4′,6-diamidino-2-phenylindole (DAPI) for nuclei.

### Statistical analysis

All the data analysis was carried out using PRISM5 (GRAPHPAD) and reported as Mean ± SEM. Statistical significance (P < 0.05) was calculated using one-way ANOVA with a *Tukey *post hoc test.

## Results and discussions

The current treatment strategies to treat bone and joint tuberculosis involve prolonged anti-tuberculosis therapy in early stages which often causes systemic toxicity^33–37^. To circumvent these limitations of poor bioavailability, various implantable ATD carriers have been developed to deliver the drugs locally at the infection site. All the hitherto ATD carriers developed, have shown burst release or were not able to achieve long term sustained release of drugs^9,13,14,38^. In the current study, we have used a ceramic based hydroxyapatite bone substitute as a carrier of two first line anti-tuberculosis drugs, Rifampicin and Isoniazid. The potency of a sulphate hydroxyapatite carrier for bioactive molecules to promote bone healing was established in earlier studies^22,26,39–41^. Apart from achieving long term sustained release of drugs, it can act as a bone filler material to fill the dead space at the debrided site. The local environment with hypoxia and clot formation in the dead space has been found to be favourable for dormant bacteria to start growing again^42^.

### Characterisation of NC and drug@NC

**Figure 1:**
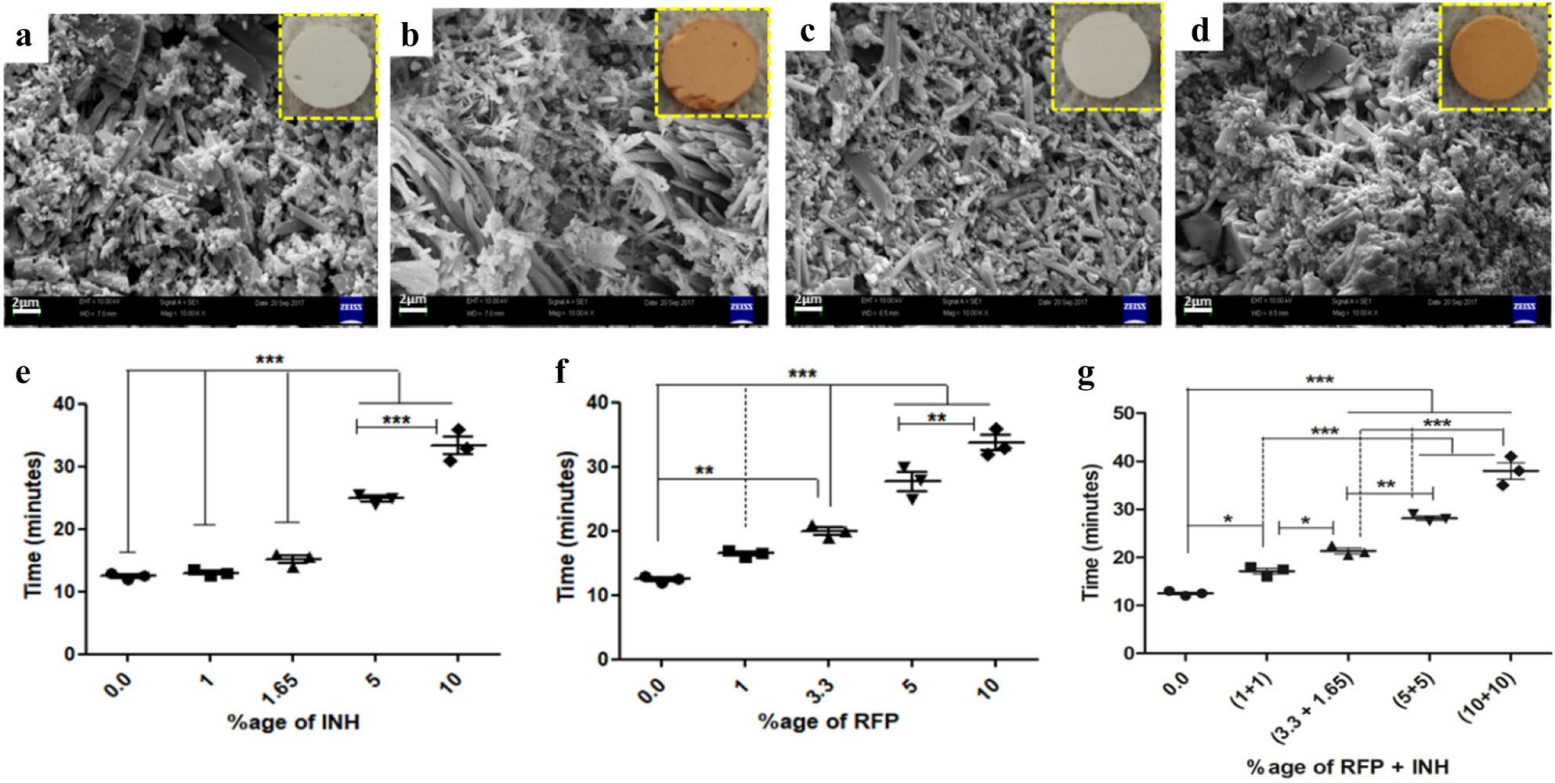
SEM images of (**a**) NC alone, (**b**) NC + RFP, (**c**) NC + INH, (**d**) NC + RFP + INH. Insets show the digital images of NC pellets loaded with RFP, INH and both. Setting time of NC with increasing concentration of drugs; (**e**) NC + INH, (**f**) NC + RFP, (**g**) NC + RFP + INH. Scale bar-2 µm & magnification- 10KX. NC-nanocement. INH-isoniazid. RFP-rifampicin. *p < 0.05, **p < 0.01, ***p < 0.001.

SEM micrographs of NC and drug@NC depicted that there was not any gross change to the microstructure of nanocement when INH or RFP alone or in combination was added to it. We observed a significant increase in the setting time of NC upon addition of drugs alone or in combination and the setting time increased with increasing concentration of drugs (Fig. 1). Moreover, the compressive strength of nanocement was observed to reduce significantly (*p* < 0.05) with increasing concentration of RFP and INH, alone or in combination as represented by peak stress (MPa) (FigureS1). These findings demonstrate that antibiotics could be incorporated in NC up to certain concentration beyond which there is a drastic change in the setting time and in other physicochemical properties and renders the carrier inefficient as a carrier.

### In-vitro drug release profile from drug@NC

**Figure 2:**
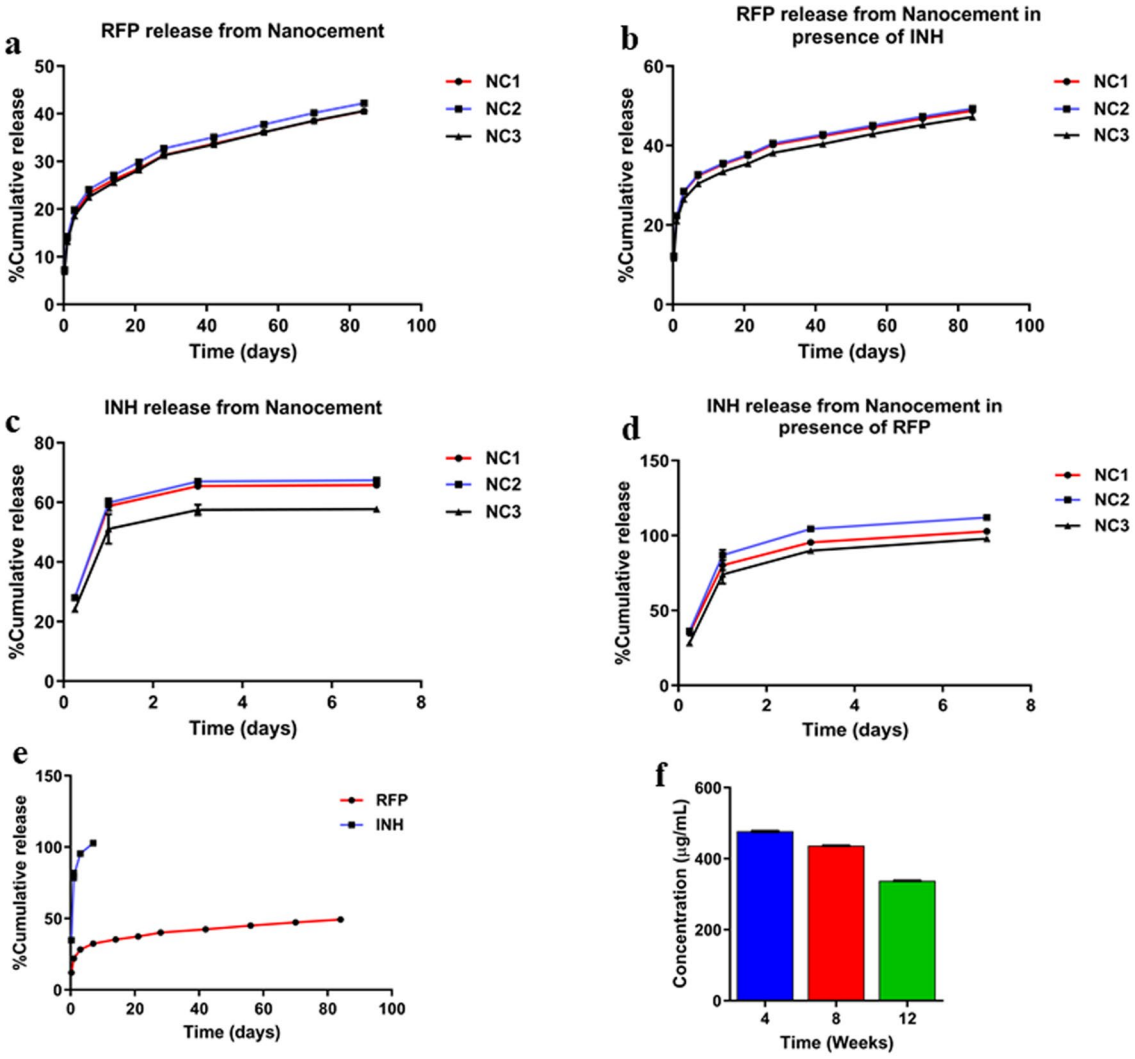
In-vitro percentage cumulative drug release profile from calcium sulphate hemihydrate (CSH)/nanohydroxyapatite (nHAP) based nanocement (NC) for 12 weeks. (**a**) RFP release from NC containing RFP (NC + RFP), (**b**) RFP release from NC containing RFP and INH (NC + RFP + INH), (**c**) INH release from NC containing INH (NC + INH), (**d**) INH release from NC containing RFP and INH (NC + RFP + INH), (**e**) RFP and INH release pattern from NC containing RFP and INH (NC + RFP + INH). (**f**) Concentration of RFP released at 4, 8 and 12 week from NC containing RFP (NC + RFP). NC1 = CSH:nHAP (80:20), NC2 = CSH:nHAP (60:40), NC3 = CSH:nHAP (40:60).

We didn’t observe any difference in the release profile of drugs from nanocement1, nanocement2 and nanocement3. In NC1, NC2 and NC3, the ratio of CSH:nHAP is different, however, the amount of RFP/INH incorporated is kept constant. The amount of nHAP matrix available for interaction and holding RFP is enough in all the ratios and it is this interaction that helps retaining the RFP for longer time and not INH. However, a different release pattern of RFP and INH was observed from nanocement wherein RFP showed sustained and controlled release over a period of 12 weeks unlike INH which showed burst release. It was observed that 42 ± 0.03% of RFP was released after 84 days when RFP alone was added to NC matrix unlike INH where 67 ± 0.01% of INH was released after one week when INH alone was mixed with nanocement, after which the concentration of INH was undetectable in UV spectrophotometer. However, we observed that when both RFP and INH were mixed with NC matrix, 100 ± 0.01% of INH was released from the matrix within one week unlike RFP which depicted sustained release and 49 ± 0.02% of RFP was released after 84 days (Fig. 2). The difference in the release pattern was attributed to the strong interaction between RFP and HAP achieving a sustained release. Moreover, the concentration of RFP released from the nanocement (NC + RFP) was calculated at 4 weeks (477.15 ± 3.03 µg), 8 weeks (436.50 ± 1.67 µg) and 12 weeks (337.67 ± 1.56 µg) time points and it was found to be higher than the minimal inhibitory concentration (MIC) of *Mycobacterium tuberculosis* (Fig. 2f). Moreover, such different interaction behaviour of two antibiotics corroborate well with the fact that dual antibiotic therapy with temporal separation can reduce the risks of emergence of drug resistant bacteria which is often seen in single antibiotic therapy^43^.

### Nanohydroxyapatite—drug interactions

#### Fourier-transform infrared (FTIR) spectroscopy

**Figure 3:**
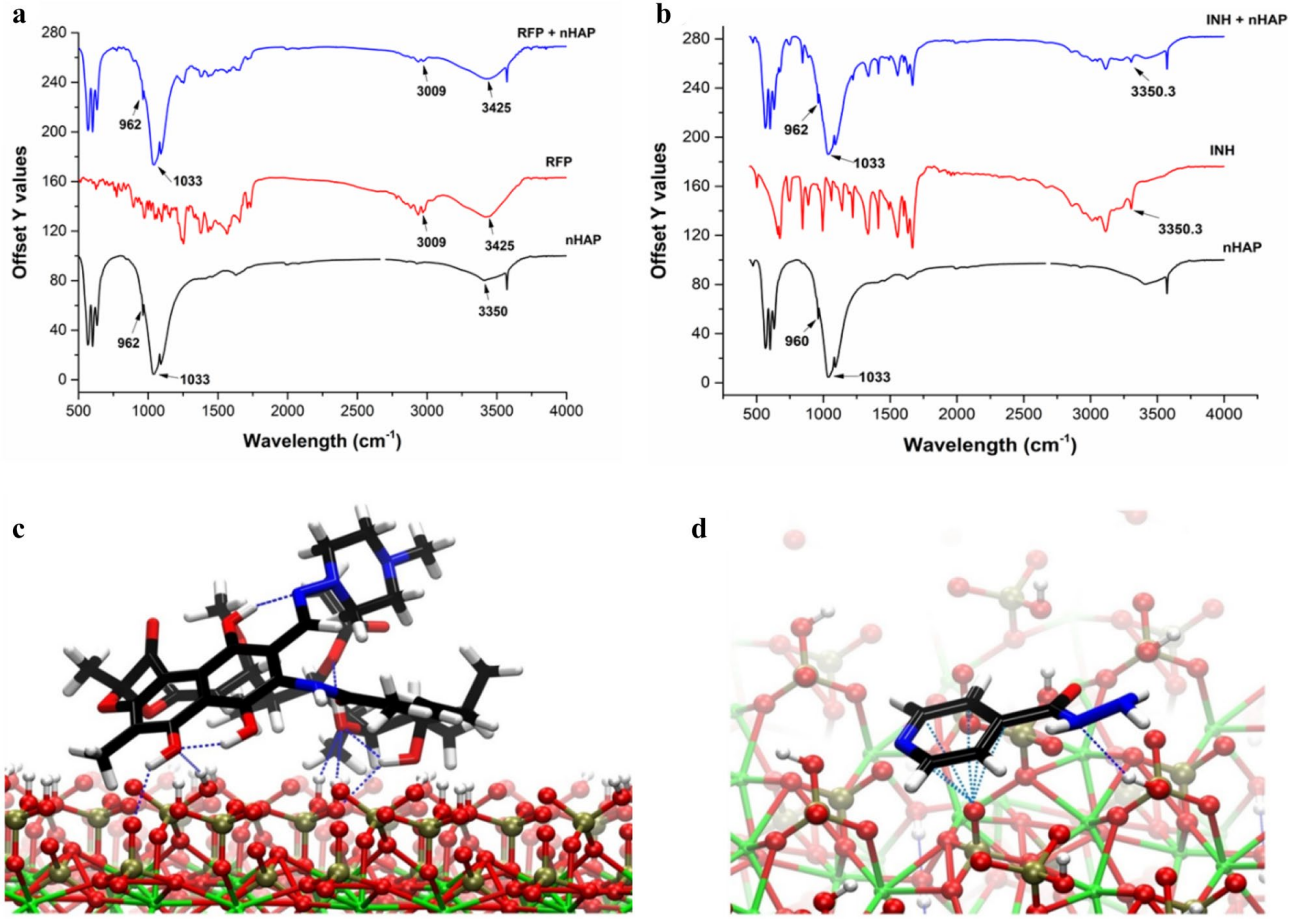
Antibiotics-NHAP Interaction: IR spectra showing interaction of RFP and INH with nHAP as indicated by specific peaks. (**a**) FTIR of RFP + nHAP, RFP and nHAP. (**b**) FTIR of INH + nHAP, INH and nHAP. (**c**) Energetically preferred adsorption structures of RFP, and (**d**) INH. Color Code: O (red), N (blue), P (ochre), Ca (green), H(white), H-boding (blue dotted lines).

The FTIR spectra showed that RFP and nHAP had a strong hydrogen bonding interaction wherein free –OH groups of phenol in RFP (on both front and rear side) make several hydrogen bonds with the free –OH groups on the surface of nHAP. In pure nHAP, we observed specific bands for PO4^3-^ in nHAP at 962 cm^−1^ and 1,033 cm^−1^. When nHAP was mixed with RFP, we observed overlapping of bands in a stretch from 3,100–3,500 cm^−1^ corresponding to adsorption of RFP onto the nHAP. Moreover, at 1,200–1,700 cm^−1^ stretch, bands particular to RFP disappeared or low intensity bands were observed. This finding is reasonable as the adsorbed molecule (RFP) has a lower degree of freedom for movement and vibration than the adsorbing surface (nHAP). Reduction in vibrations at wavelength 2,936–3,009 cm^−1^ (C=C–H in aromatic compounds and –OH of phenol in RFP) in nHAP-RFP shows that the phenolic –OH group of RFP is now hydrogen bonded to nHAP (Figure S2). The overlapping of peaks in nHAP + RFP from 3,000 cm^−1^ to 4,000 cm^−1^ show that free –OH groups in nHAP is now hydrogen bonded to RFP (Fig. 3a). Further the data was also supported by deconvulation of FTIR spectra as shown in supplementary information (Figure S3). In case of INH, we again observed the overlapping of band and thus adsorption of INH on nHAP. The band stretching was observed at 3,350 cm^−1^ only that is attributed to N–H stretching in INH and thus suggesting that N–H in INH and free –OH of nHAP are involved in hydrogen bonding (Fig. 3b).

### Computational studies

The (highest) binding energies of RFP and INH on the surface of nHAP are 148 kcal/mol and 11 kcal/mol, respectively. Stability of the structure of nHAP-RFP and nHAP-INH systems depend on the number of hydrogen bonding interactions (intermolecular/intramolecular) present in the system as well as the strength of these hydrogen bonds. In the most stable structures of nHAP-RFP system, four intermolecular (i.e. substrate-adsorbate) and four intramolecular hydrogen bonds are present (Fig. 3c). The hydroxylated phosphate moieties from hydroxyapatite take part as hydrogen bonding donor as well as acceptor. Surface oxygen atoms are also participating in hydrogen bonding with the hydroxyl groups of the drug molecule. The second lowest energy structure (not shown here), which is ~ 5 kcal/mol higher in energy than the most stable structure, has three intermolecular and five intramolecular hydrogen bonds. Although the number of hydrogen bonds is higher in this structure compared to the lowest energy structure, the orientation of the piperazine ring in the former structure entails weaker intramolecular hydrogen bonds. This could explain the energy difference observed for the two structures.

In the case of the lowest energy conformation of the nHAP-INH system, the planar pyridine ring of the adsorbate is slightly bent towards the surface. Only one adsorbate–substrate hydrogen bond is present here (which is between Nitrogen atom in the drug and the surface hydroxyl in nHAP), while a lone pair (lp) – π type interaction between the π cloud of the aromatic pyridine ring of INH and the lp of oxygen in the surface PO_4_ moiety (Fig. 3d) could potentially provide additional stability. No intramolecular hydrogen bond is present, unlike in the case of nHAP-RFP.

Based on the structural analysis of the most stable conformations of adsorbate molecules on nHAP surface, we infer that the difference in the interactions between the adsorbate and the surface hydroxyl moieties is responsible for the higher binding energy of RFP on nHAP. The IR spectra of the bare surface, adsorbates, and the adsorbed complexes are given in Figure S4. The IR spectra for the RFP/INH adsorbed on nHAP, in range 3,000–4,000 cm^−1^, are showing overlapping of bands, in qualitative agreement with the experimental FTIR spectrum.

### Bactericidal effect

**Figure 4:**
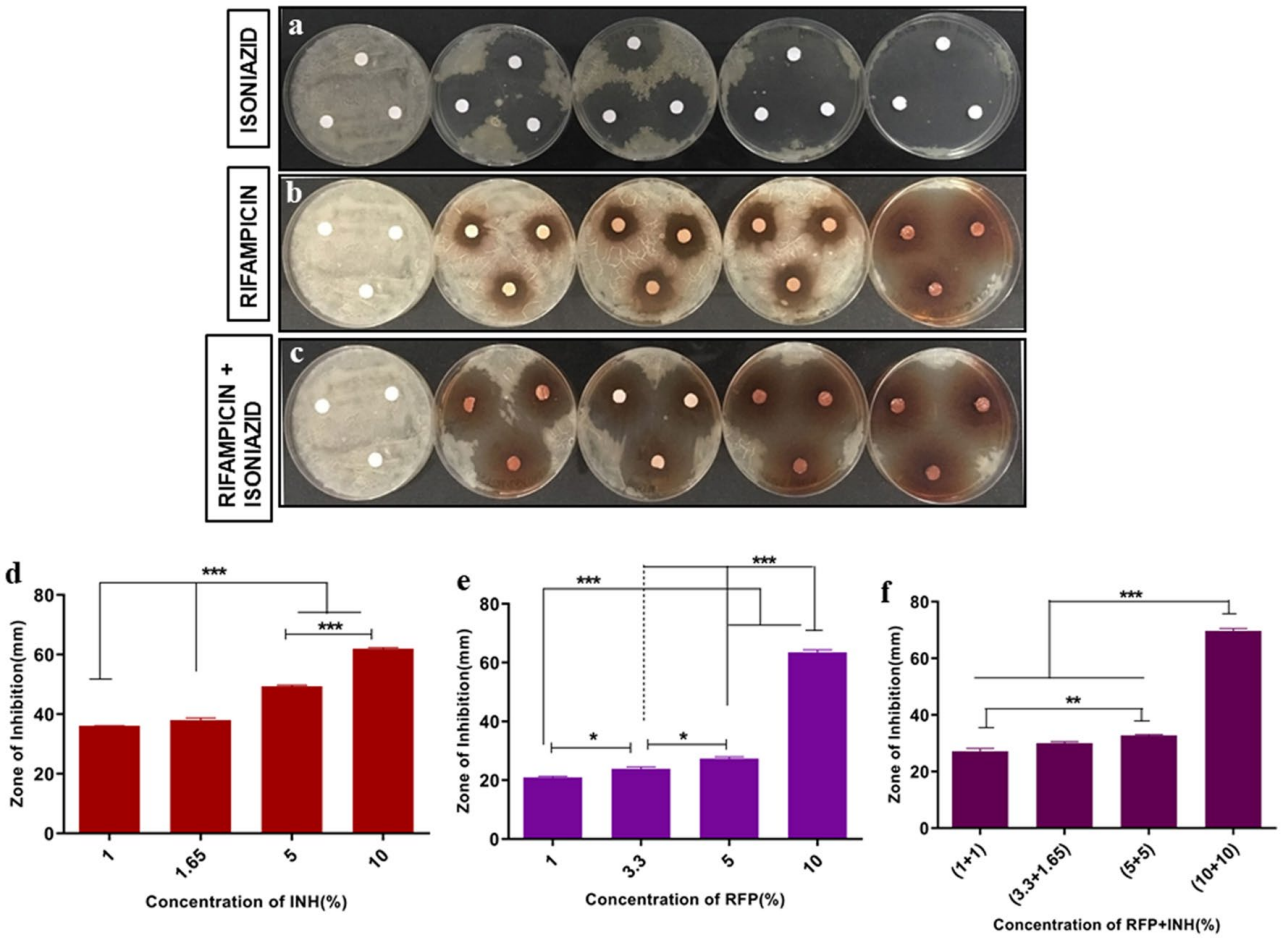
Kirby-Bauer (KB) disk diffusion assay. Agar diffusion assay of calcium sulphate hemihydrate (CSH)/nanohydroxyapatite (nHAP) based nanocement (NC) and drug loaded NC (drug@NC) showing anti-mycobacterial (*Mycobacterium smegmatis*) activity of pristine released RFP and INH as indicated by increased zone of inhibition (ZOI) surrounding drug@NC. Digital picture of NC loaded with increasing concentration of (**a**) INH (1%, 1.65%, 5% & 10%). (**b**) RFP (1%, 3.3%, 5% & 10%) and (**c**) RFP + INH (1% + 1%, 3.3% + 1.65%, 5% + 5% & 10% + 10%). Quantification of ZOI using digital Vernier calliper in NC loaded with increasing concentration of (**d**) INH, (**e**) RFP, (**f**) RFP + INH. *p < 0.05, **p < 0.01, ***p < 0.001.

**Figure 5:**
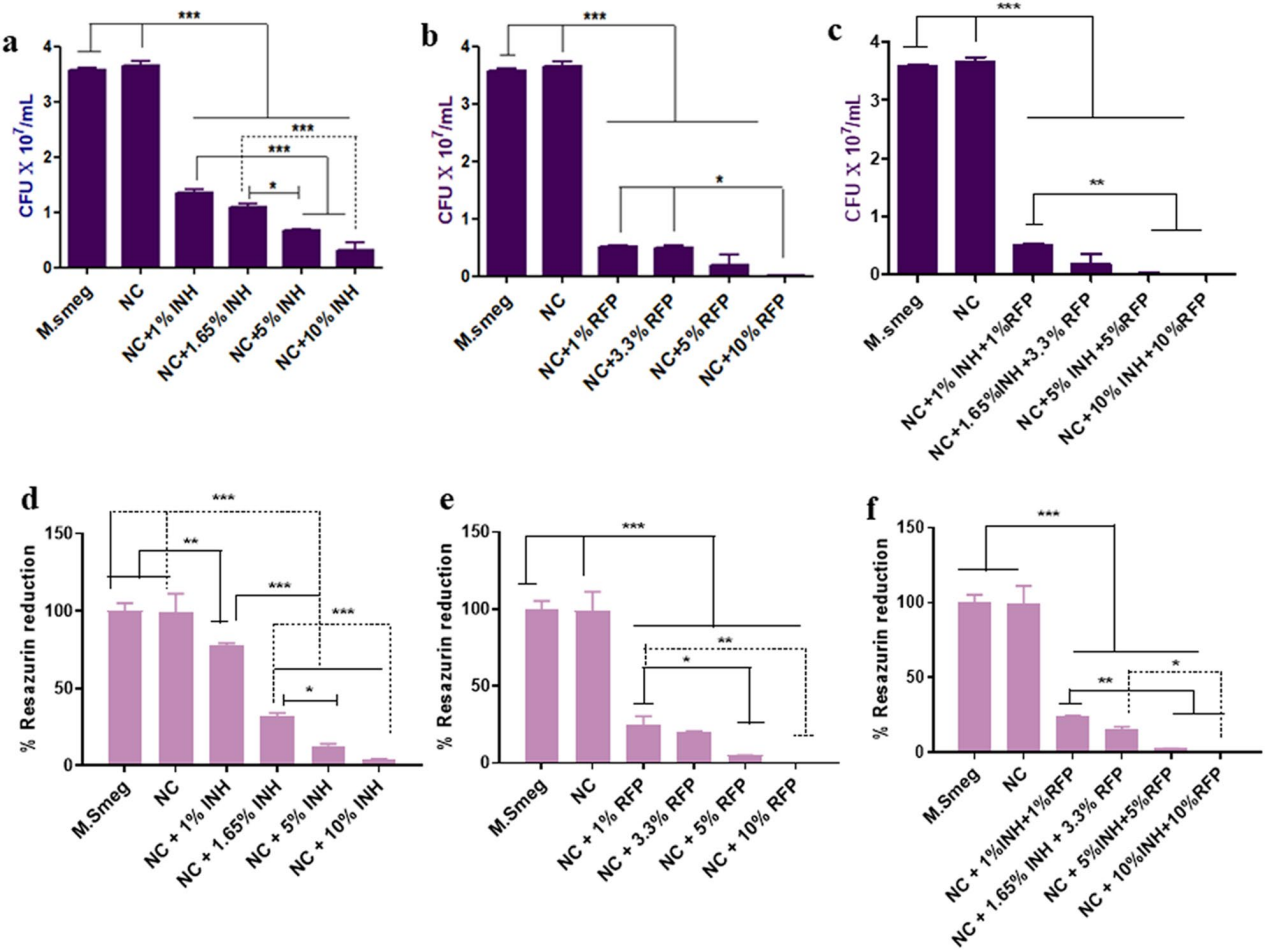
Antibacterial activity of calcium sulphate hemihydrate (CSH)/nanohydroxyapatite (nHAP) based nanocement (NC) and drug loaded NC (drug@NC). Liquid inoculation assay of drug loaded NC with increasing concentration of drugs by counting the colony forming units (CFU). (**a**) INH, (**b**) RFP & (**c**) RFP + INH. Resazurin assay showing enhanced antibacterial activity of drug loaded NC (drug@NC) by measuring percentage resazurin reduction. (**d**) INH, (**e**) RFP & (**f**) RFP + INH. *p < 0.05, **p < 0.01, ***p < 0.001.

It is imperative that the drug is released at MIC levels, effective to kill the pathogens. We have shown that the released drugs (RFP and INH) showed dose dependent inhibition of bacterial growth in *Mycobacterium smegmatis* as seen in agar diffusion assay (Fig. 4), liquid inoculation assay and resazurin assay. The zone of inhibition (mm) surrounding NC was observed to increase significantly (*p* < 0.05) with increasing concentration of drugs (RFP and INH, alone or in combination). We observed that the CFU count reduced significantly (*p* < 0.05) while increasing the concentration of drugs (RFP and INH alone or in combination) in the nanocement matrix. The CFU count in M. smegmatis (control) and nanocement was significantly higher when compared to all the concentrations of RFP, INH and RFP + INH used in NC (Fig. 5). Resazurin reduction assay was used to determine the viable count of bacteria present when exposed to NC and drug@NC. The enzymes in the metabolically active cells will reduce the reazurin to resorufin that can be measured flourimetrically. We observed that *M. smegmatis* and NC alone showed significantly enhanced resazurin reduction when compared to all other concentration of INH, RFP and RFP + INH incorporated into NC (Fig. 5).

### Antibiofilm assay

**Figure 6:**
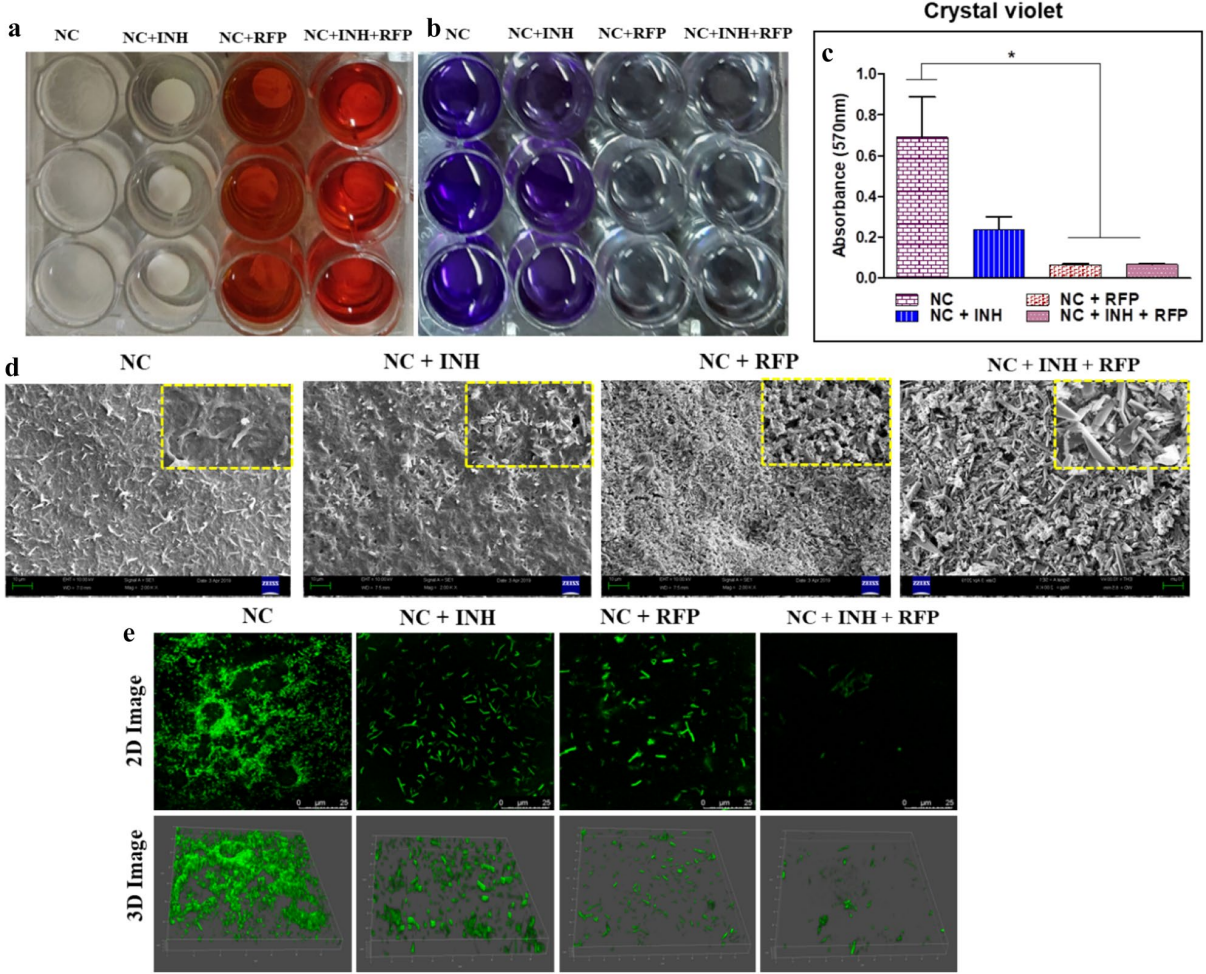
Antibiofilm assay. *Mycobacterium smegmatis* was allowed to grow undisturbed on calcium sulphate hemihydrate (CSH)/nanohydroxyapatite (nHAP) based nanocement (NC) and drug loaded NC (drug@NC) to induce biofilm formation. (**a**) Digital picture of a plate wherein NC and drug@NC show biofilm formation and inhibition, respectively. (**b**) Digital picture of a plate for crystal violet in which the antibiofilm assay was carried out. (**c**) Quantification of crystal violet assay. (**d**) Scanning electron micrograph (SEM) images of *M. smegmatis* biofilm formation on the surface of NC and drug@NC. (**e**) Confocal laser scanning microscopy (CLSM) analysis of biofilm inhibition by drug loaded calcium sulphate hemihydrate (CSH)/nanohydroxyapatite (nHAP) nanocement (drug@NC). Both 2D and 3D images show proper biofilm formation on the surface of NC and not on the surface of NC + INH, NC + RFP and NC + RFP + INH as observed by GFP expression. drug@NC = NC + INH, NC + RFP and NC + RFP + INH. *p < 0.05, **p < 0.01, ***p < 0.001.

**Figure 7:**
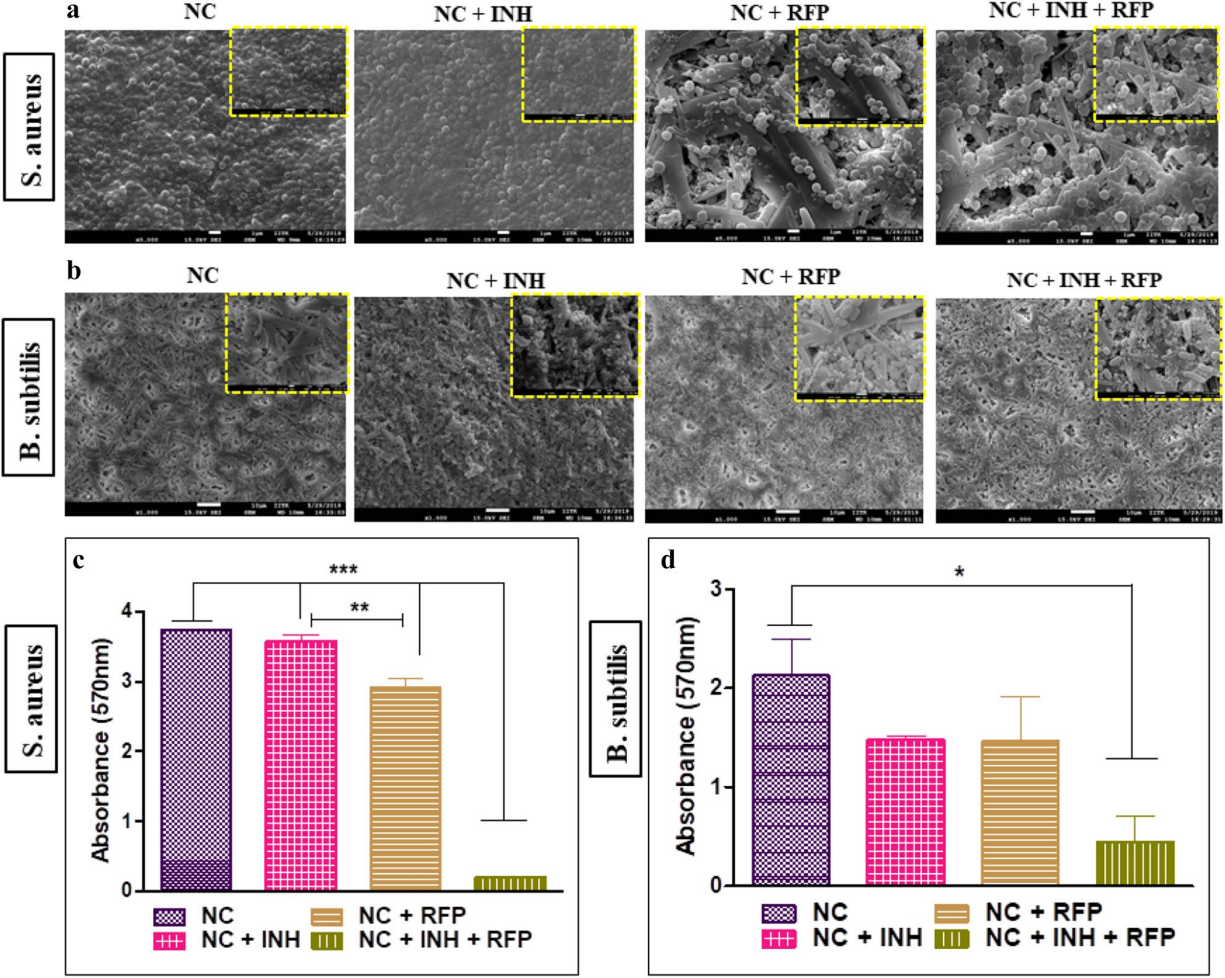
Antibiofilm assay was carried out and visualized by scanning electron micrograph (SEM) images by growing (**a**) *Staphylococcus aureus* and (**b**) *Bacillis subtilis* undisturbed on the surface of calcium sulphate hemihydrate (CSH)/nanohydroxyapatite (nHAP) based nanocement (NC) and drug loaded NC (drug@NC). The plates were further analysed for crystal violet assay (**c**) *S. aureus* and (**d**) *B. subtilis*. drug@NC = NC + INH, NC + RFP and NC + RFP + INH. *p < 0.05, **p < 0.01, ***p < 0.001.

Biofilm associated infection in orthopaedic implants is one of the major concerns using biomedical devices^44,45^. We used three reference bacteria; *M. smegmatis, S. aureus* and *B. subtilis*. It was demonstrated that NC + RFP + INH combination is highly active in inhibiting the biofilm formation by these pathogens, which might allow osteoblasts to migrate to the surface and thus prevent the pathogens from adhering to the surface. In case of *M. smegmatis*, we observed that only RFP or combination of RFP and INH could inhibit the formation of biofilm significantly (*p* < 0.05) as observed in SEM micrographs and crystal violet quantification. In presence of NC + INH, the biofilm formation was not hampered significantly when compared to NC alone (Fig. 6). The CLSM analysis of inhibition of biofilm formation on the surface of drug@NC also corroborated with the fact that drug impregnated nanocement precludes the formation of biofilm on its surface. We observed only planktonic bacteria on the surface of drug@NC with higher number on NC + INH and lesser number on NC + RFP and NC + RFP + INH (Fig. 6). The biofilm formation in case of *S. aureus* on the surface of NC and drug@NC was observed to get inhibited significantly when both RFP and INH was added to nanocement when compared to NC, NC + INH and NC + RFP. Moreover, NC + RFP also depicted significant inhibition of biofilm formation as compared to NC + INH. However, in case of *B. subtilis*, the inhibition of biofilm formation was found to be significantly affected by NC + RFP + INH when compared to NC or NC + INH or NC + RFP (Fig. 7).

#### Cell material interactions

**Figure 8:**
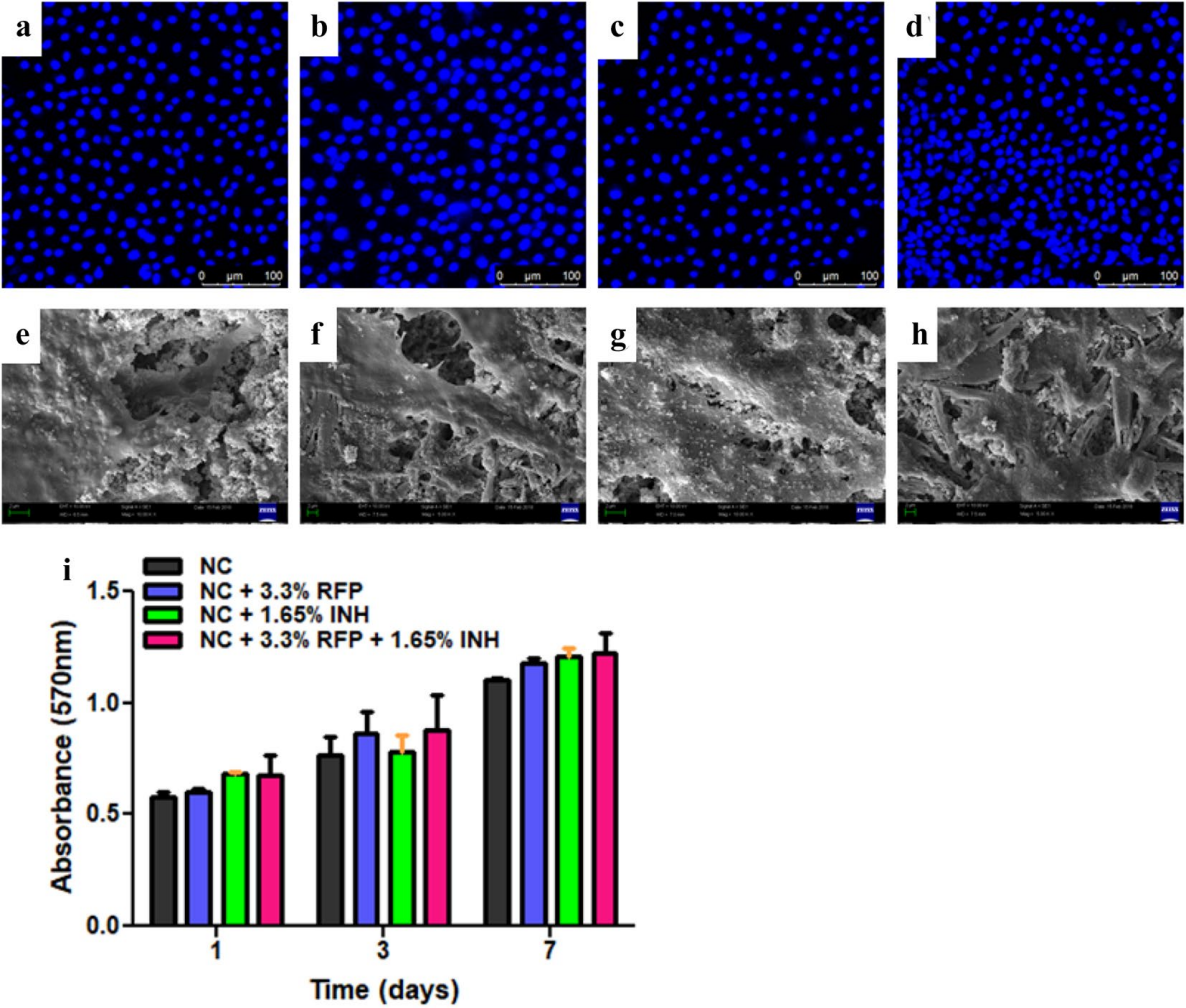
Cell material interactions. Murine pre-osteoblasts (MC3T3E1) grown on the surface of calcium sulphate hemihydrate (CSH)/nanohydroxyapatite (nHAP) based nanocement (NC) and drug loaded NC (drug@NC) for seven days. DAPI staining of cells at day 7 grown on (**a**) NC, (**b**) NC + INH, (**c**) NC + RFP and (**d**) NC + RFP + INH**.** Scanning electron micrograph (SEM) images of cells at day 7 grown on (**e**) NC, (**f**) NC + INH, (**g**) NC + RFP and (**h**) NC + RFP + INH, (**i**) MTT assay for seven days showing cell proliferation of cells and non-cytotoxicity of materials.

To investigate cell material interactions and biocompatibility of drug laded nanocement, murine pre-osteoblast cells (MC3T3E1) were allowed to grow on the NC and drug@NC. The cell adhesion was evaluated by SEM micrographs that showed proper and uniform distribution of cells at day 7 over the matrices. Moreover, numerous filipodial extensions and anchoring appendages were observed in all directions. Further, the cell adhesion was also confirmed by CLSM by staining with DAPI at day 7. The cell proliferation was assessed by MTT assay for 7 days and it was observed that both NC and drug@NC showed increased cell proliferation from day1 to day7 (Fig. 8).

### In-vitro Mycobacterium smegmatis infection

**Figure 9:**
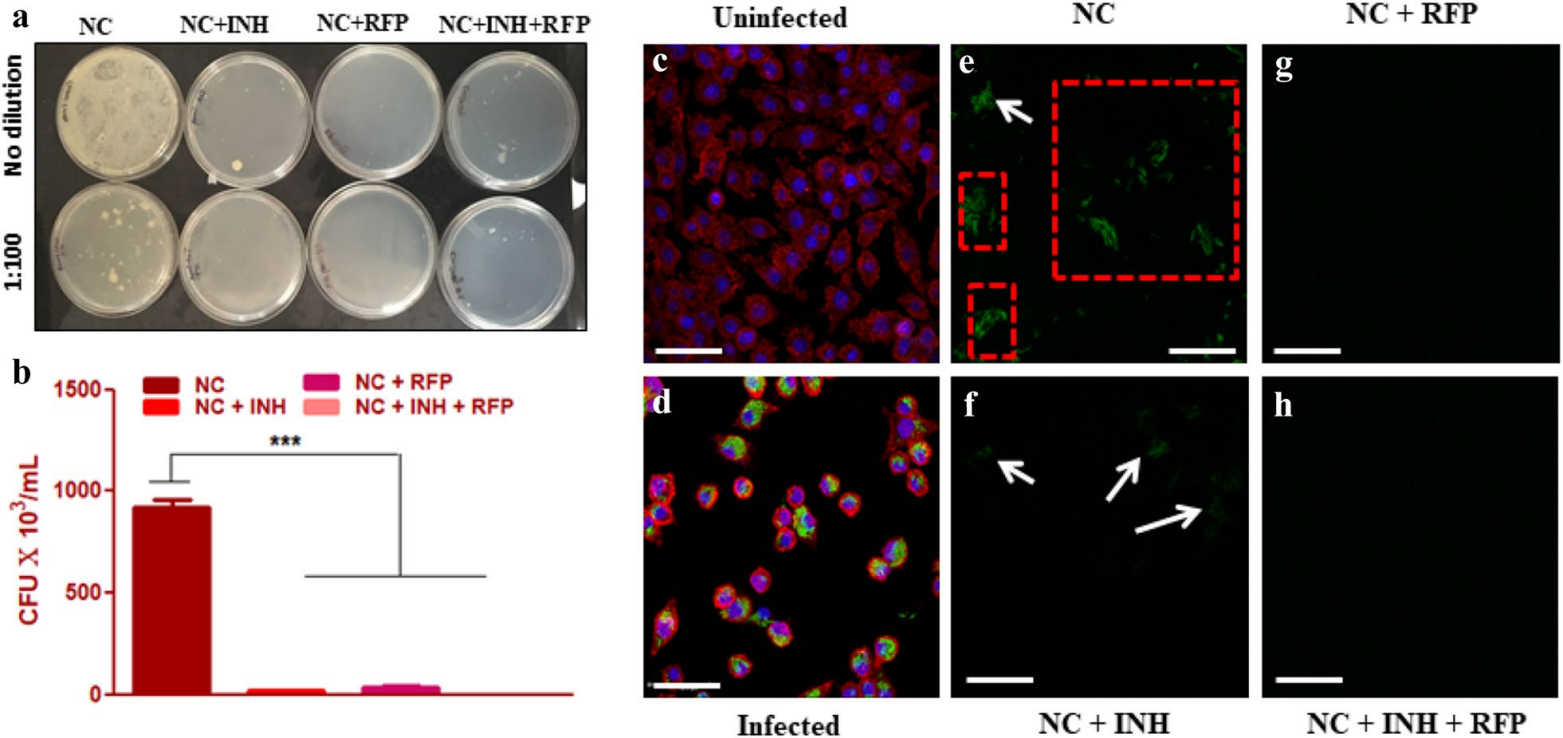
In-vitro *Mycobacterium smegmatis* infection. RAW 264.7 murine macrophages were grown on NC & drug loaded NC (drug@NC) and infected with *M. smegmatis* followed by collection of cell lysate for CFU count. (**a**) Digital picture of agar plates with dilution to see the CFU count on the plates. (**b**) CFU count of bacteria in cell lysate of infected cells in drug impregnated NC (drug@NC) when compared to NC. (**c**) Uninfected macrophages and (**d**) infected macrophages on the coverslips. (**e**) GFP expression on the surface of NC 24 h post-infection of macrophages (white arrow and red boxes). (**f**) GFP expression on NC + INH 24 h post-infection showing very little GFP expression as indicated by white arrows, (**g**), (**h**) GFP expression on the surface of NC + RFP and NC + RFP + INH, respectively showing no GFP expression. Scale bar = 50 µm. drug@NC = NC + INH, NC + RFP & NC + RFP + INH. *p < 0.05, **p < 0.01, ***p < 0.001.

*Mycobacterium* is an intracellular pathogen and often evades the endo-lysosomal pathway of the cells and thus evading the active macrophage-associated antibacterial activities. We observed that in cell lysate of infected RAW 264.7 murine macrophages, the CFU count was significantly higher (*p* < 0.05) in NC when compared to drug@NC (Fig. 9a,b). Moreover, the CLSM images on coverslips showed successful infection of macrophages as observed by intracellular presence of GFP expressing *M. smegmatis* in infected macrophages (Fig. 9d) when compared to uninfected macrophages (Fig. 9c). The GFP expression found on the surface of NC (Fig. 9e) depicted that the intracellular pathogen has not been eliminated and killed after 24 h while as a little GFP expression was observed on NC + INH (Fig. 9f) and no expression on NC + RFP (Fig. 9g) and NC + RFP + INH (Fig. 9h) showing complete elimination of intracellular pathogen from the infected macrophages.

## Conclusion

In conclusion, this study showed that a sulphate hydroxyapatite carrier could be used for local delivery of tuberculosis drugs in a controlled and sustained manner. Local drug delivery using a ceramic carrier could reduce and at best alleviate the side effects of RFP and INH systemic administration and hinder the recurrence of infection. The new findings of interaction between hydroxyapatite and RFP open up a new window of using such systems for long term drug delivery. Moreover, RFP released from hydroxyapatite matrix still have the capacity to kill he targeted bacteria at 3 months. By reducing the risk of reinfection, a hydroxyapatite based delivery system could also act as bone filler in the dead space, promoting bone regeneration.

## Supplementary information

Supplementary Information.
