# Supplementary material for: A biphasic nanohydroxyapatite/calcium sulphate carrier containing Rifampicin and Isoniazid for local delivery gives sustained and effective antibiotic release and prevents biofilm formation
Source: Sci Rep. 2020 Aug 24;10:14128. doi: 10.1038/s41598-020-70726-3 (PMC7445265; doi:10.1038/s41598-020-70726-3)
Supplement: Supplementary file 1 — Supplementary Information. [file 41598_2020_70726_MOESM1_ESM.pdf]

A biphasic nanohydroxyapatite/calcium sulphate carrier containing Rifampicin and Isoniazid for local delivery gives sustained and effective antibiotic release and prevents biofilm formation

Irfan Qayoom[1], Rahul Verma[4], Prem Anand Murugan[1], Deepak Bushan Raina[5], Arun Kumar Teotia[1], Saravanan Matheshwaran[1,2], Nisanth N. Nair[4], Magnus Tägil[5], Lars Lidgren[5], Ashok Kumar[1,2,3,*]

[1]*Department of Biological Science and Bioengineering;* [2]*Centre for Environmental Sciences and Engineering;* [3]*Centre for Nanosciences and* [4]*Department of Chemistry, Indian Institute of Technology Kanpur, Kanpur-208016, UP, India*

[5]*Department of Orthopaedics, The Medical Faculty, Clinical Sciences Lund, Lund University, Sweden*

*Correspondence should be addressed to

Ashok Kumar
Department of Biological Sciences and Bioengineering
Indian Institute of Technology Kanpur,
Kanpur-208016, UP, India
Email: ashokkum@iitk.ac.in
Phone: +91-512-2594051

Supplementary Information

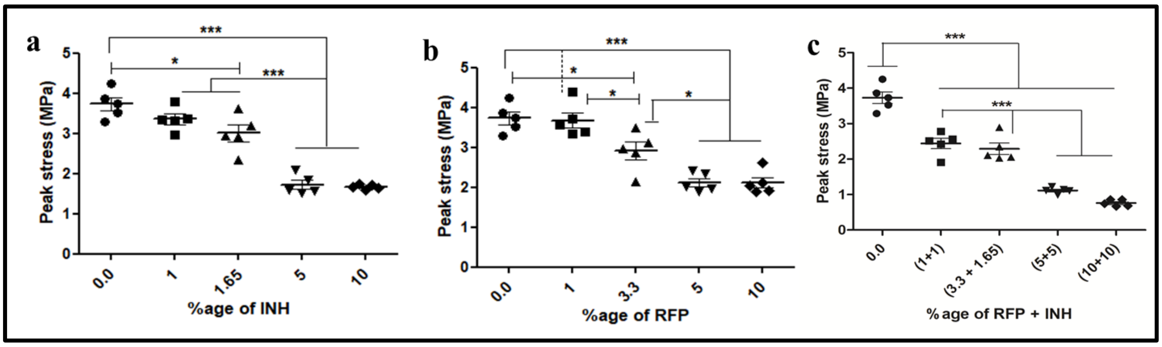

Figure S1: Compressive strength of NC with increasing concentration of drugs, **a)** NC + INH, **b)** NC + RFP & **c)** NC + RFP + INH.
NC-nanocement. INH-isoniazid. RFP-rifampicin. $^*p<0.05$, $^{**}p<0.01$, $^{***}p<0.001$.

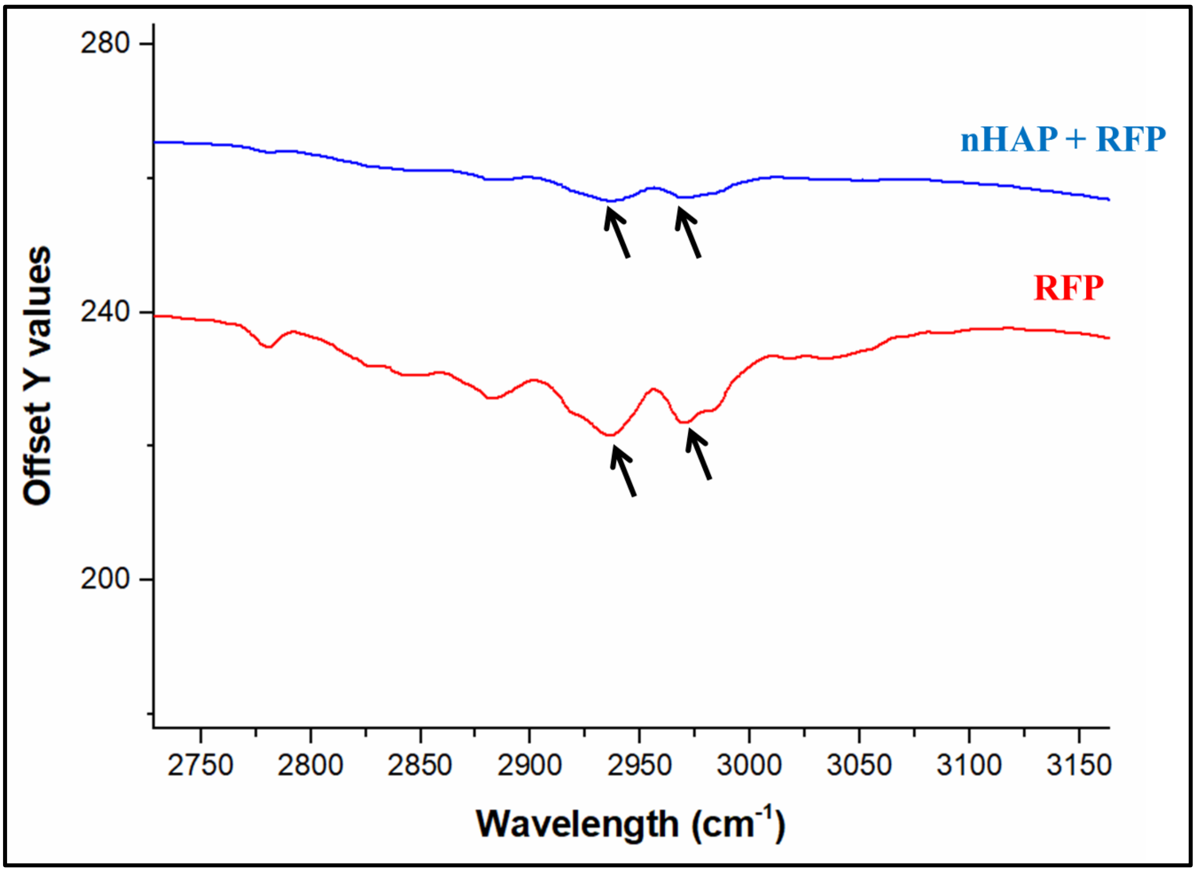

Figure S2: FTIR spectra of **a)** RFP and **b)** RFP+nHAP showing reduction in vibrations at wavelength from 2936-3009 cm^{-1} in RFP+nHAP when compared to RFP. Black arrows show the peaks at which there is reduction in the vibrations in nHAP + RFP when compared to RFP.

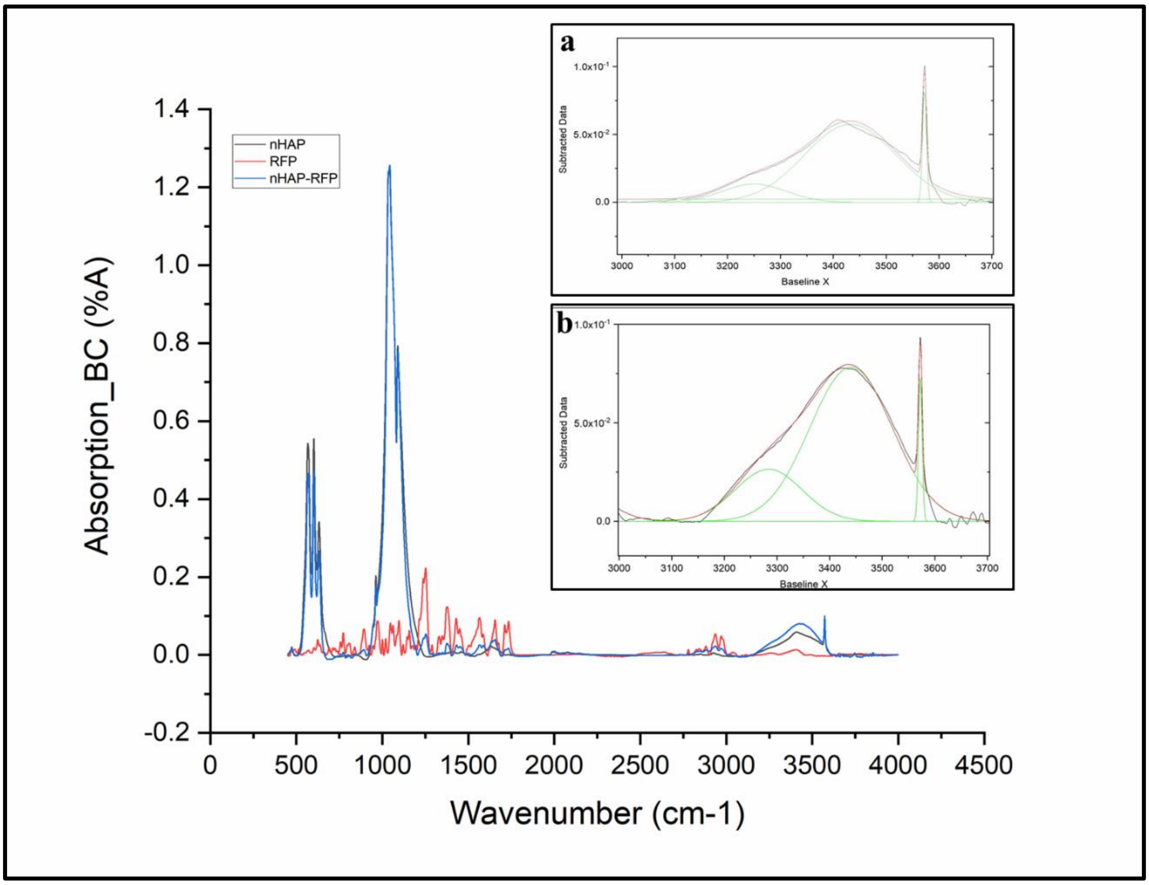

Figure S3: FTIR spectra of nHAP, RFP and nHAP incorporated with RFP with insets showing deconvoluted spectra of **a)** nHAP and **b)** nHAP + RFP from wavelength 3300 cm^{-1} to 3700 cm^{-1}.

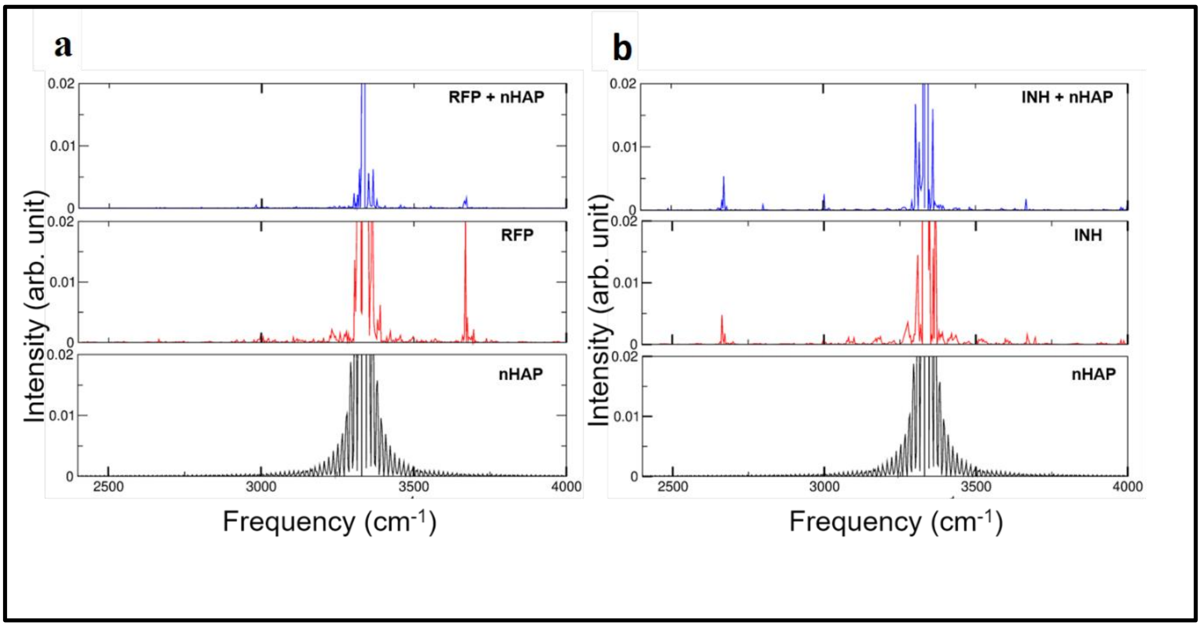

Figure S4: IR spectra computed from molecular dynamics simulations are shown here: **a)** RFP + nHAP, RFP, and nHAP; **b)** INH + nHAP, INH, and nHAP.
